# Night eating syndrome subtypes: differences in binge eating and food addiction symptoms

**DOI:** 10.1007/s40519-023-01534-7

**Published:** 2023-02-08

**Authors:** Brenda Echeverri, Andrea T. Kozak, Daniel J. Gildner, Scott M. Pickett

**Affiliations:** 1 Center for Translational Behavioral Science, Florida State University College of Medicine, Tallahassee, FL 32310 USA; 2 Department of Psychology, Oakland University, Rochester, MI 48309 USA

**Keywords:** NES typology, Eating pathology, Binge eating, Food addiction, Evening hyperphagia, Nocturnal ingestions

## Abstract

**Purpose:**

The purpose of the current study was to examine differences in binge eating and food addiction symptoms between Night Eating Syndrome (NES) latent subtypes: evening hyperphagia with nocturnal ingestions (EHNI), evening hyperphagia-only (EHO), and nocturnal ingestions-only (NIO). It was hypothesized that the EHNI group would report more binge eating behaviors and more food addiction symptoms than both the EHO and NIO groups. Further, it was hypothesized that the EHO and NIO groups would differ with the EHO group reporting more binge eating behaviors and the NIO group reporting more food addiction symptoms.

**Methods:**

Participants completed measures online relating to night eating, binge eating, and food addiction. Average age of the final sample was 34.3 (SD = 10.5) and 62.0% were men. Responses to the Night Eating Questionnaire (NEQ; Allison et al., 2008) were used to create an EHNI group (*n* = 65), an EHO group (*n* = 32), and a NIO group (*n* = 69). ANOVAs were conducted to examine between-group differences on disordered eating symptoms.

**Results:**

Participants in the EHNI group reported more severe binge eating and food addiction symptoms than those in the EHO and NIO groups. However, there were no significant differences in binge eating or food addiction between the EHO and NIO groups.

**Conclusion:**

Individuals who meet both NES core criteria (evening hyperphagia and nocturnal ingestions) are likely at a higher risk for experiencing other, more severe disordered eating pathologies. Implications concerning assessment and future research on NES typology are discussed.

**Level of evidence:**

Level V, cross-sectional descriptive study.

## Introduction

There has been an increased interest concerning Night Eating Syndrome (NES) in the last decade as it is related to a multitude of behavioral and psychological disorders. NES is included in the fifth edition of the Diagnostic and Statistical Manual of Mental Disorders (DSM-5) as an unspecified eating disorder with two core diagnostic criteria symptoms, evening hyperphagia (i.e., consuming 25% or more of total daily caloric intake after the evening meal) and episodes of consuming food upon awakenings during the night, called nocturnal ingestions [1, 2]. Other suggested NES symptom criteria may include morning anorexia, insomnia, and evening mood deterioration [2–4]. Allison and colleagues (2010) proposed that NES core symptoms—namely, evening hyperphagia and nocturnal ingestions—could potentially be representative of NES subtypes, but further investigation is required [3].

Even though only an estimated 1.5% of the general population is diagnosed with NES [5], prevalence rates escalate considerably among clinical samples, such that 22.4–25.0% of psychiatric outpatients meet criteria for NES [6–8]. Moreover, NES is highly comorbid with other eating pathology [9], undesired weight gain and increased risk of obesity [10], poor sleep quality [11, 12], anxiety [4, 13, 14], depressive symptoms [15–18], and poor overall mental health [19]. Due to high rates of comorbidity, NES diagnostic criteria remain broad and controversial and NES assessment is often overlooked in clinical settings [20]. Additional research on NES nosology is warranted to provide a better understanding of NES and improve assessment, intervention, and preventative measures.

NES typology has hardly been explored and that is most likely because there has not been a clear consensus regarding potential NES subtypes. Based on evening hyperphagia, nocturnal ingestions, and depressed mood symptoms, Striegel-Moore et al. (2008) categorized individuals who reported night eating as follows: individuals who ate a significantly large amount of food in the evening (between 7:00PM and 11:00PM) with and without depressed mood and those who ate late at night (after 11:00PM) with and without depressed mood. In 2010, an international NES research group concluded that depression was not required for diagnosis and should only be considered as an optional NES feature [3, 21]. In fact, depression is not included in the DSM-5 description of NES [1]. Thus, it was proposed that NES subtypes be examined based on an individual consuming more than 25% of total daily calories after the evening meal (i.e., evening hyperphagia) and/or experiencing night eating episodes upon awakening from sleep (i.e., nocturnal ingestions), suggesting three potential NES subtypes [3].

Since the NES international meeting [3] and the publication of the DSM-5 [1], the authors are aware of only one study that has examined differences between NES subtypes [22]. Loddo et al. (2019) screened participants for NES symptom criteria and created two NES subgroups, an evening hyperphagia (with/without nocturnal eating episodes) group and a nocturnal ingestions-only group. The evening hyperphagia (with/without nocturnal ingestions) group consisted of 2 participants who reported evening hyperphagia without nocturnal ingestions and 8 participants who reported evening hyperphagia with nocturnal ingestions. The nocturnal ingestions-only subgroup consisted of 10 participants who experienced nocturnal ingestions, but no evening hyperphagia [22]. Participants’ sleep and eating episodes were examined via video-polysomnography and significant differences were found concerning nocturnal eating behaviors between the two NES subgroups [22]. Notably, individuals in the evening hyperphagia subgroup had longer waking episodes and spent more time engaging in other activities (other than eating) before consuming food, after having eaten a snack, and before going back to sleep, than those in the nocturnal ingestions-only subgroup [22]. These initial data suggest that evening hyperphagia and nocturnal ingestions may be the framework of NES subtypes. However, distinctions between evening hyperphagia with and without nocturnal ingestions were not analyzed. It would be beneficial to examine differences between NES subtypes to better understand NES symptomology and improve NES assessment.

Given that eating patterns appear to be different between those who experience evening hyperphagia and those who only report nocturnal ingestions [22], it is important to address whether NES subtypes differ in terms of eating pathology symptoms. For example, bingeing behavior is generally observed in evening hyperphagia [23], but it does not appear to occur during nocturnal ingestions [22]. Therefore, more severe binge eating symptoms would be expected among individuals who fall within the evening hyperphagia subtype compared to those in the nocturnal ingestions subtype. Furthermore, individuals who report both, evening hyperphagia and nocturnal ingestions, are likely to represent a more severe subtype*.* Despite symptomology similarities between NES and binge eating disorder (BED), significant differences have been observed. For example, individuals with obesity and NES have more difficulty losing weight than those with obesity and BED [24]. NES and BED symptoms are frequently correlated [4, 25], but it seems conceivable that bingeing behaviors would be more relevant to evening hyperphagia and not as pertinent to a nocturnal ingestions-only subtype.

Calories consumed at night typically consist of foods that are carbohydrate-based or high in fat and sugar [26, 27]. Accruing data suggest that highly processed foods such as desserts, potato chips, and pastries, might trigger eating behaviors that mirror addictive-like characteristics, a phenomenon known as food addiction [28]. Food addiction is a topic of much controversy, but nonetheless relevant to NES research because of the types of foods consumed at night. Food addiction symptoms are positively correlated with NES severity, and more specifically, food tolerance (a symptom of food addiction) may be a significant predictor of NES severity [29]. However, whether food addiction is (or which food addiction symptoms are) associated with evening hyperphagia, nocturnal ingestions, or both remains unknown.

### Hypotheses

To the authors’ knowledge, no previous studies have examined disordered eating behavior differences in relation to NES subtypes. The current study aimed to examine differences in self-reported binge eating symptoms and food addiction symptoms between possible NES subtypes, defined a priori according to the NES typology suggested by Allison and colleagues (2010). Binge eating symptoms and food addiction symptoms were examined among three NES subgroups: an evening hyperphagia with nocturnal ingestions group (EHNI), an evening hyperphagia-only group (EHO), and a nocturnal ingestions-only (NIO) group. Based on previous data [29], greater overall NES scores are expected to be positively correlated with more binge eating symptoms and food addiction symptoms. It is hypothesized that the EHNI group will display more severe bingeing behaviors and more food addiction symptoms than the EHO and NIO groups. Similarly, it is hypothesized that bingeing behaviors and food addiction symptoms will be significantly different between the EHO and NIO groups, with the EHO reporting more bingeing behaviors and the NIO group reporting more food addiction symptoms.

## Method

### Participants

Participants were recruited through Amazon’s Mechanical Turk (MTurk), a suitable platform to recruit a more diverse sample than a convenience sample would provide [30]. Participants had to be at least 18 years old, be able to read and understand English, and provide consent prior to completing the survey study online. All participants answered the questionnaires in the same order and demographic questions were presented at the beginning of the survey. Incomplete responses, meaning entirely incomplete questionnaires relevant to the current study, led to being excluded from the study. Participants who reported a complete lack of awareness when getting up in the middle of the night to snack, which would signal a different nocturnal eating problem (i.e., parasomnia Sleep-Related Eating Disorder), were also excluded. Participants who did not provide height or weight, or who fell in the underweight body mass index (BMI) category were excluded from the study. BMI and age outliers were identified as outliers using boxplot analyses and were excluded from final analyses. Extreme BMI outliers comprised three individuals with a BMI = 56.5 kg/m^2^, 58.0 kg/m^2^, and 78.3 kg/m^2^. The final sample consisted of 63 (38.0%) women and 103 (62.0%) men. A larger proportion of men in the current study was not surprising. Generally, NES affects women and men at relatively similar rates according to a review [25] and National Health and Nutrition Examination Survey (NHANES-III) data suggest that men are slightly more likely to consume food later in the evening and at night [31]. Upon survey completion, participants were compensated with $1.50 for their efforts. All procedures were approved by the University’s Institutional Review Board (1204014-6).

### Measures

#### Night eating questionnaire

The Night Eating Questionnaire (NEQ) has been widely used to examine NES symptoms [32]. Comprising 14 items, the NEQ is answered on a 4-point Likert scale, each item with different response options that correspond to the question. Sample items include: “Do you have cravings or urges to eat snacks after supper, but before bedtime?” and “How much of your daily food intake do you consume after suppertime?” [32]. Stop criteria are built into the measure such that if the respondent indicates no nocturnal awakenings or not snacking in the middle of the night, items that pertain to those specific behaviors are not shown to the respondent [32]. One item is included in the measure to screen out individuals who lack awareness when snacking in the middle of the night, a condition otherwise known as parasomnia Sleep-Related Eating Disorder (SRED) [32]. NEQ global scores range from 0 to 52 (does not include SRED item). The NEQ has demonstrated adequate reliability (Cronbach’s alpha = 0.70), discriminant validity (among patients with obesity), and convergent validity [32]. In the current study, the standardized alpha was 0.66 for the NEQ in the current study.

#### Binge eating scale

The Binge Eating Scale (BES) assesses behavioral and cognitive aspects of binge eating [33]. The BES has 16 items, each consisting of three to four statements in which the respondent selects the one they feel best describes them. Sample items include: “I don’t feel guilt or self-hate after I overeat,” “After I overeat, occasionally I feel guilt or self-hate,” and “Almost all the time I experience strong guilt or self-hate after I overeat.” Each possible response is assigned a score from 0 to 3 and the sum of all responses ranges from 0 to 46. A continuous score is often used in analyses; however, clusters of severity may be categorized to reflect the following: no binge eating problems (total score less than 17), moderate binge eating problems (total score between 18 and 26), or severe binge eating problems (total score of 27 or higher) [33]. The BES has demonstrated discriminant validity and satisfactory test–retest reliability (*r* = 0.87) according to another study [34]. In the current study, the BES demonstrated a Cronbach’s alpha of 0.91.

#### The yale food addiction scale

The Yale Food Addiction Scale (YFAS 2.0) was developed to screen for addictive-like behaviors toward foods that are typically high in fats and refined sugars [35]. Addictive-like behaviors were defined in accordance with the DSM-5 substance-related and addictive disorders [1]. The YFAS 2.0 consists of 35 questions with different scoring thresholds. Sample items include “When I started to eat certain foods, I ate much more than planned” and “I really wanted to cut down on or stop eating certain kinds of foods, but I just couldn’t.” Responses are recorded on a scale from 0 = *never* to 7 = *every day*. The YFAS 2.0 yields a continuous symptom count (i.e., food addiction symptoms) and probable clinical diagnosis [35]. Convergent, discriminant, and incremental validity as well as good internal reliability (Kuder–Richardson alpha of 0.90) were reported for the YFAS 2.0 [35]. The total symptom count is obtained based on whether individual items meet the threshold for each criterion and the final scores are considered dichotomous (absent or present). In the current study, the internal consistency for the YFAS 2.0 was good (Kuder–Richardson = 0.95).

### NES groups

In accordance with NES typology research [3, 22], three groups were created in the current study: an EHNI (i.e., evening hyperphagia and nocturnal ingestions) group, an EHO (i.e., evening hyperphagia, no nocturnal ingestions) group, and a NIO (i.e., nocturnal ingestions, no evening hyperphagia) group. Using the NEQ, evening hyperphagia was defined as consuming more than 25% of total daily calories (i.e., NEQ item 5: “How much of your daily food intake do you consume after suppertime?”). Similarly, nocturnal ingestions were inferred if participants reported eating a snack (at least sometimes) in the middle of night (i.e., NEQ item 12: “When you get up in the middle of the night, how often do you snack?” [32]. Data from 334 participants were used to create the three NES subgroups; participants who did not meet criteria for evening hyperphagia nor indicated experiencing nocturnal ingestions (*n* = 168) were excluded from the rest of the study. The EHNI group consisted of 65 participants, the EHO consisted of 32 participants, and the NIO only consisted of 69 participants. These sample sizes were expected given prevalence rates in the general population and that the sample was not a clinically representative sample. The NES groups in the current study were distributed at relatively equal rates as those in the study by Loddo and colleagues (2019), in which the group with individuals who only experienced evening hyperphagia (and no nocturnal ingestions) was undersized compared to the other NES subgroups. Correspondingly, in the current study, there were fewer participants who fell within the EHO group than participants in the EHNI and NIO groups.

### Statistical methods

Participants were grouped into either an EHNI group (*n* = 65), an EHO group (*n* = 32), or a NIO group (*n* = 69). More specific NES group composition is outlined in Table 2. Prior to conducting analyses, both outcome variables were assessed for normality with both binge eating (skewness = -0.09, kurtosis = -0.84) and food addiction symptoms (skewness =  – 0.19, kurtosis =  – 1.62) appearing to follow a normal distribution. Analyses of variance (ANOVAs) were then conducted to examine between-group differences on binge eating symptom severity and food addiction symptoms. A Pearson’s Chi-Square test was then utilized to test the effect of the NES groups on probable food addiction diagnosis. All analyses were conducted using SPSS (v.26) and alpha values *p* < 0.05 were considered statistically significant.

## Results

### Participant demographics

A total of 166 eligible participants were included in the study analyses to test the study hypotheses. The mean age for the final sample was 34.3 (*SD* = 10.5). Almost half of the sample (*n* = 80; 48.2%) reported a BMI (kg/m^2^) in the normal range, 30.1% (*n* = 50) had a BMI in the overweight range, and 21.7% (*n* = 36) had a BMI in the obese range. The majority of the sample (*n* = 120; 72.3%) were white or Caucasian, 12.7% (*n* = 21) were Black or African American, 7.2% (*n* = 12) were Asian, 6.0% (*n* = 10) were American Indian or Alaskan Native, and 3 participants indicated “other” as their race. Of the total sample, 12.0% were Hispanic/Latinx. 30 (18.1%) participants reported having at least a graduate degree, 75 (45.2%) participants had at least a four-year degree, 49 (29.5%) reported having completed some college (e.g., two-year degree), and 12 (7.2%) participants had a high school diploma only. Prior to testing the main hypotheses, descriptive statistics and bivariate correlations were examined and are presented in Table 1.

**Table 1:** Bivariate correlations, means, and standard deviations for study variables

Variables	1	2	3	4	5	6	7	8	9
1. Age in years	–								
2. Sex (% Male)	– 0.16*	–							
3. Race (% White)	0.24**	– 0.15	–						
4. BMI	0.02	– 0.02	0.09	–					
5. Food addiction symptom count	– 0.11	0.10	– 0.10	– 0.24**	–				
6. Binge eating symptoms	– 0.07	– 0.08	– 0.03	0.07	0.56**	–			
7. Night eating global	– 0.04	– 0.11	– 0.14	0.00	0.47**	0.54**	–		
8. NEQ item 5	– 0.06	0.10	– 0.03	0.14	0.19*	0.22**	0.23**	–	
9. NEQ item 12	– 0.17*	0.06	– 0.02	– 0.31**	0.44**	0.36**	0.59**	0.07	–
*Mean*	34.28	0.62	4.40	26.31	5.87	15.54	20.94	1.75	1.39
*SD*	10.53	0.49	1.20	5.41	4.36	10.09	6.17	0.78	1.03

Sex (% Male), 0 = Female/1 = Male; Race (% White), 0 = All races other than White/1 = White; Counseling Exposure: “How long have you been or were you in counseling or therapy?”, 0 = No/1 = Yes; Food addiction = food addiction symptom count; NEQ item 5: “How much of your daily food intake do you consume after suppertime?”; NEQ item 12: “When you get up in the middle of the night, how often do you snack?”

^*^p < .05, **p < .001

### Group differences

#### Binge eating

**Fig. 1:**
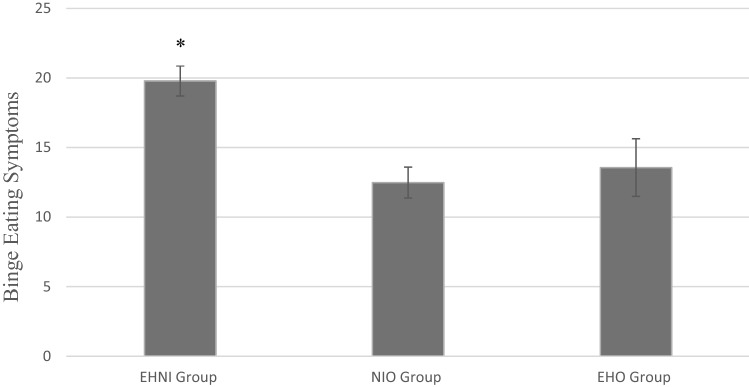
Binge eating symptom severity across night eating groups. *Note*. Error bars reflect standard error of the mean. * Reflects group significantly differed from other groups. *EHNI* = evening hyperphagia and nocturnal ingestions; *NIO* = nocturnal ingestions only; *EHO* = evening hyperphagia only

An ANOVA revealed a significant difference between the three groups on binge eating symptom severity; *F*_(2, 163)_ = 10.66, *p* < 0.001, *η*^2^ = 0.12. However, results from the Levene’s test suggest that the homogeneity of variance assumption had been violated indicating that the variances between the groups were not relatively equal. As such, a non-parametric multiple comparison procedure, Games–Howell, was implemented to account for the unequal variances between the groups [36]. Post hoc comparisons revealed that the EHNI group (*M* = 19.79, *SD* = 8.67) reported more binge eating symptoms than all other groups, while the NIO (*M* = 12.48, *SD* = 9.24) and EHO (*M* = 13.56, *SD* = 11.70) groups did not significantly differ from each other. Table 2 further illustrates these post hoc comparisons and Fig. 1 provides a visual depiction.

**Table 2:** NES subgroup composition and post hoc comparisons between night eating subgroups

	1. EHNI (*n* = 65)		2. EHO (*n* = 32)		3. NIO (*n* = 69)				
	*n*	%	*n*	%	*n*	%	CHI-SQUARE	EFFECT SIZE (Cramér’s V)	SIG
Sex (Male)	41	63.1	22	68.8	40	58.0	*χ*^2^_2,166_ = 1.13	0.08	NS
Race (White)	49	75.4	23	71.9	48	69.6	*χ*^2^_2,166_ = .569	0.75	NS

	*M*	*SD*	*M*	*SD*	*M*	*SD*	ANOVA	EFFECT SIZE (η^2^)	POSTHOC
Mean Age (*SD*)	33.43	10.51	34.31	9.52	35.07	11.05	0.40*	0.005	NS
Mean BMI (*SD*)	25.15	4.20	29.42	7.12	25.96	5.03	7.46***	0.084	2 > 1,3
Food Addiction Symptoms	7.48	3.58	3.38	3.48	4.35	4.08	18.13**	0.180	1 > 2,3
Binge Eating Symptoms	19.79	8.67	13.56	11.7	12.48	9.24	10.66***	0.120	1 > 2,3

*EHNI* evening hyperphagia and nocturnal ingestions; *EHO* evening hyperphagia-only; *NIO* nocturnal ingestions-only; *NS* non-significant

^*^p > .05, df(2,163)

^**^p < .001, df(2,162)

^***^p < .001, df(2,163)

#### Food addiction

**Fig. 2:**
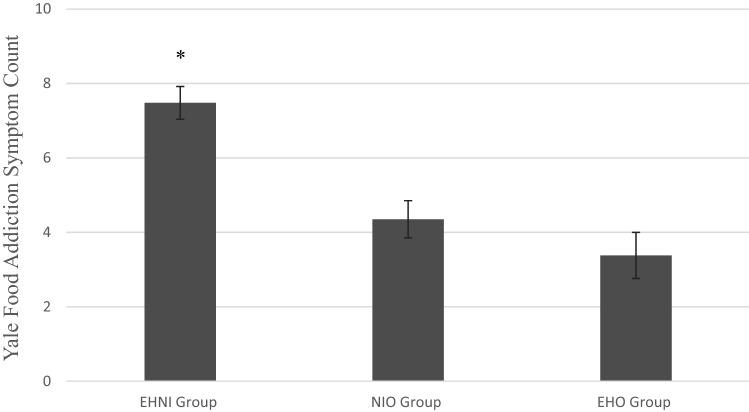
Food addiction symptoms across night eating groups. *Note*. Error bars reflect standard error of the mean. * Reflects group significantly differed from other groups. *EHNI* = evening hyperphagia and nocturnal ingestions; *NIO* = nocturnal ingestions only; *EHO* = evening hyperphagia only

There was a significant difference between the three night eating groups on food addiction symptoms; *F*_(2, 162)_ = 18.13, *p* < 0.001, *η*^2^ = 18. Similar as above, the homogeneity of variance assumption had been violated and the Games–Howell procedure was implemented. The results of the post hoc comparisons suggest that the EHNI group (*M* = 8.12, *SD* = 3.52) reported more food addiction symptoms than all other groups, while the NIO (*M* = 4.76, *SD* = 4.44) and the EHO (*M* = 3.66, *SD* = 3.75) groups did not significantly differ from each other. Table 2 further illustrates these post hoc comparisons and Fig. 2 provides a visual depiction.^1^ According to the symptom severity scoring option [35], 38 participants in the EHNI group, 6 participants in the EHO group, and 18 participants in the NIO group met suggested clinical criteria for food addiction diagnosis (i.e., clinical distress and at least 3 other food addiction symptoms).

#### Exploratory analyses

An exploratory analysis was conducted to examine the effect of the symptom severity scoring option [35] on the night eating group subtypes. The suggested clinical criteria for food addiction diagnosis (i.e., clinical distress and at least 3 other food addiction symptoms) served as the criteria to create a no food addiction (*n* = 97) and a probable food addiction (*n* = 69) group. A 3 (NES groups) X 2 (probable food addiction diagnosis) Pearson’s Chi-Square Test of Independence was then conducted to examine whether the distribution of scores were independent of each other. The results of this test suggest a significant association between the groups, *X* ^2^(2, *N* = 166) = 18.45, *p* < 0.001 to a strong degree (Cramér’s *V* = 0.33, *p* < 0.001).

For the group with a probable food addiction (*n* = 69), 58.0% were in the EHNI group while 31.9% and 10.1% fell in the NIO and EHO groups, respectively. Alternatively, for those who did not meet the food addiction threshold (*n* = 97), the pattern of results was not as distinguished: 48.4% of participants were in the NIO group and 25.8% and 25.8% were in the EHO and EHNI groups, respectively. These results suggest that comparatively, the EHNI group self-reported more instances of probable food addiction diagnosis, while a clear pattern of results could not be determined from those without a probable food addiction diagnosis.

Additionally, a second exploratory analysis was conducted to examine the effect of binge eating symptom severity [33] on the night eating subtypes. Three independent groups were created based on the respondents sum of binge eating symptom severity. A no binge eating problem group (*n* = 80), a moderate binge eating problem group (*n* = 66), and a serious binge eating problem group (*n* = 20) [33]. A 3 (NES groups) X 3 (binge eating groups) Pearson’s Chi-Square Test of Independence was then conducted to examine whether the distribution of scores were independent of each other. The results suggest a significant association between the groups, *X* ^2^(4, *N* = 166) = 22.60, *p* < 0.001 to a strong degree (Cramér’s *V* = 0.26, *p* < 0.001).

For the group with no binge eating problems (*n* = 80), 55.0% were in the NIO group while 23.8% and 21.2% fell in the EHO and EHNI groups, respectively. Furthermore, for the moderate binge eating group (*n* = 66), 57.6% were in the EHNI group, 30.3% were in the NIO group, and the remaining 12.1% were in the EHO group. Lastly, for individuals within the serious binge eating problem group (*n* = 20), 50% of participants fell within the EHNI group, 25.0% were within the NIO group, and the remaining 25.0% were in the EHO group. These results suggest that comparatively, individuals with moderate to serious binge eating symptom severity were most likely to fall within the EHNI group.

## Discussion

The literature concerning NES typology remains meager. In 2010, Allison and colleagues (2010) highlighted the need for a more detailed examination of three possible NES subtypes based on evening hyperphagia and/or nocturnal ingestions [3]. However, latent NES subtypes have not been thoroughly explored and more research is warranted to inform this area of NES [3]. Additional research on NES typology could inform future research studies for diagnosis and treatment of NES. To the authors’ knowledge, only one study has examined differences among potential NES subtypes regarding sleep patterns [22], but no studies have examined disordered eating symptom differences. The current study informs the question concerning NES subtypes and differences in eating pathology, specifically as it relates to binge eating and food addiction symptoms. Using the NEQ items that concentrate on evening hyperphagia and frequency of nocturnal ingestions, three NES groups were created: an EHNI group, which consisted of individuals who met criteria for evening hyperphagia and also reported nocturnal ingestions; an EHO group consisting of individuals who met criteria for evening hyperphagia but indicated that they never consumed snacks during sleep awakenings; and a NIO group, consisting of individuals who reported nocturnal ingestions, but did not meet criteria for evening hyperphagia.

The hypothesized differences among NES subgroups were partially supported by the results. Overall, the EHNI group reported more severe binge eating and more food addiction symptoms than the EHO and NIO groups. The EHO and NIO groups did not differ from each other in terms of binge eating severity or food addiction symptoms. These results suggest that a NES subtype in which both core criteria are met (i.e., EHNI) may be indicative of more severe binge eating and food addiction symptoms than in the case in which only one of the two NES core criteria exist (i.e., EHO or NIO). The hypothesis regarding differences in binge eating symptoms between the EHO and NIO groups was not supported, which was unexpected given that evening hyperphagia seems to be more specific to binge eating pathology [23, 37] and nocturnal ingestions are not characterized by bingeing behavior [22]. Thus, binge eating symptoms were expected to be more prevalent among the EHO group in comparison to the NIO group, but the results from this study did not support this notion.

Food addiction symptoms and NES symptoms were significantly correlated in the current study as in previous studies [29, 38]. Moreover, individuals who were in the EHNI group reported more food addiction symptoms on average than those in the EHO and NIO groups. This suggests that individuals who report both evening hyperphagia and nocturnal ingestions are more likely to report more food addiction symptoms than individuals who only report one of the two NES core criteria. Exploratory analyses also revealed that more participants (61.3%) in the EHNI group with probable food addiction diagnosis (i.e., clinical distress and at least 3 other food addiction symptoms) [35] displayed more food addiction symptoms than did participants with probable food addiction in the EHO and NIO groups. The EHO and NIO groups did not differ in terms of food addiction symptoms. Accordingly, reporting NES symptoms that would fit within an EHNI subtype may raise a concern for probable and more severe food addiction diagnosis in an individual than reporting EHO or NIO alone.

There are a few possible explanations as to why the EHO and NIO groups did not show significant differences regarding binge eating or food addiction symptoms. One aspect is the use of self-report measures in this study, which may not capture the most accurate representation of binge eating and food addiction symptoms, rendering a follow-up clinical interview worth considering in future studies. Regarding food addiction symptoms, even though both EHO and NIO groups had a similar frequency of food addiction symptoms, it is possible that the specific symptoms that each group reported may have been different. This was beyond the scope of the purpose of the current study, however, future studies may consider exploring this question. Additionally, one must bear in mind that other cognitive and behavioral differences not included in the current study may exist between an evening hyperphagia-only subtype and a nocturnal ingestions-only subtype. For example, compared to patients that report evening hyperphagia with/without nocturnal ingestions, patients who only meet the nocturnal ingestions criterion seem to have longer nocturnal eating episodes and it takes them longer to fall back asleep [22]. NES is also characterized by evening mood deterioration, e.g., [39], and some data suggest that NES patients who experience nocturnal ingestions report more severe depression symptoms than those without nocturnal eating episodes [16]. Unfortunately, researchers have only compared NES patients with nocturnal ingestions to those without, thus failing to differentiate between subtypes concerning evening hyperphagia with and without nocturnal ingestions. Additional research examining possible differences among all three latent NES subtypes is warranted.

Research on disordered eating behaviors related to obesity is essential to inform preventative strategies and treatment. Multiple factors appear to influence the link between NES and BMI, such that some studies have found a direct association, while others, including the current study, have not (see [40] for review). Still, symptoms of NES can be expected to (at least) contribute to excess weight. For example, evening hyperphagia is common among individuals with obesity [41] and nocturnal eating is associated with significant weight gain [42]. In addition to other, more severe eating pathology that might be present [23], NES patients are undoubtedly at high risk for developing and maintaining obesogenic behaviors and metabolic syndrome [40]. The multifactorial nature of NES demands several preventative and treatment options. Specifically, lifestyle modification that includes a balanced, nutrient-dense diet, increased physical activity, and modifying the individual’s environment are some of the most well-known obesity preventative strategies [43]. It is also important to note that obesity and NES preventative strategies may start as early as in childhood and adolescence [44, 45]. NES treatment has received more attention in clinical research than preventative strategies have, however, obesity prevention may serve as a safeguard for development or worsening of NES and comorbid eating pathology (notably BED and food addiction symptoms). More research is needed in this area, especially for an EHNI subtype which may require more intensive treatment than the single NES criterion subtypes*.*

Among U.S. adults, obesity has continually increased in the last decade with obesity rates rising to 42.4% [46]. Obesity is linked to an increased risk of a variety of serious medical conditions along with premature death and reduced health-related quality of life, making effective treatment imperative [47, 48]. It is crucial to examine eating behaviors that contribute to significant weight gain in the first place [10] given the obesity epidemic in the U.S. and many other countries [49]. Behavioral weight loss treatment involves the use of cognitive behavioral therapy (CBT) [e.g., self-monitoring, social support, cognitive restricting] to lower caloric consumption and increase physical activity. This gold standard treatment can result in a weight reduction of up to 10% of body weight [50]. However, there is ample room for improvement because only around 20% of individuals enrolled in this type of treatment program are able to lose this amount of weight and keep it off for at least one year [51]. One possibility to explain the weight loss struggle is disordered eating comorbidity. When both evening hyperphagia and nocturnal ingestions are present, assessing for binge eating and/or food addiction symptoms would be beneficial in clinical research and practice. On the other hand, assessing for binge eating and food addiction symptoms may not be as crucial if only one of the two NES core criteria are present. If it is determined that a client meets the threshold of both evening hyperphagia and nocturnal ingestions, then behavioral weight loss treatment could be supplemented with treatment targeting NES symptoms such as pharmacotherapy (e.g., sertraline, escitalopram) [40]. Non-pharmacological treatments are also an option such as progressive muscle relaxation, bright light therapy, or CBT for NES which has consisted of sleep hygiene, healthy nutrition strategies, and psychoeducation [40]. CBT for NES seems particularly promising given that behavioral weight loss treatment already incorporates the use of CBT.

Finally, it is important to consider the mechanisms that underlie problematic eating behavior. For example, problems with obesity and overeating are often linked to a history of trauma [32, 52, 53] and its sequalae, such as PTSD symptoms and poor emotion regulation, e.g., [27, 54]. Another example is emotional eating, or the tendency to overeat in response to negative affect, which appears to play a significant role in the association between night eating and BMI as well as binge eating [9]. Clinical NES diagnosis may be associated with lack of appetite in the morning, insomnia, problematic beliefs about eating to aid sleep, and mood deterioration in the evening [25]; thus, future research should explore other psychopathological differences that may exist between NES subtypes and which mechanisms may influence those relationships.

### Strengths and limitations

The findings of the current study must be interpreted in the context of certain limitations. The absence of experimental manipulation and the cross-sectional nature of the data do not allow for any conclusions about causation between NES and other eating pathology. Additionally, data were collected online from U.S. based MTurk workers and should be interpreted with caution as these data are self-reported and can compromise quality of data [55]; however, attention checks, ensuring only one survey was completed per IP address, and collecting data from Mturk workers with at least 95% approval rate were used as safeguards to increase the quality of data [56]. BMI was calculated using self-reported weight and height which could have contributed to the significant negative relationship between food addiction and BMI as previous studies have usually found food addiction to be present in individuals who are categorized as obese [57]. It might not be feasible from a financial perspective to measure weight and height, but future research should examine the other variables from this study using methodology such as clinical interviews. More specifically, future food addiction research would likely benefit from the development of a reliable and valid semi-structured interview because a self-report questionnaire is the only validated tool at the current time. Another limitation was that the sample consisted mostly of Caucasian, non-Hispanic participants, which makes it difficult to know whether the results would generalize to individuals with a different racial or ethnic background. Future studies should aim to recruit and compare groups of equal size because it could reveal important group differences. Similarly, researchers may consider including a control group to compare to NES subtypes. Despite the differences in group sizes in the current study, a strength of the current study is a larger sample than what Loddo et al. (2019) used to compare NES subtypes. In the current study, using the NEQ had limitations, including the moderate standardized alpha and the inability to screen for shift workers. Future studies may benefit from using a categorical instrument for NES, such as the Night Eating Diagnostic Questionnaire (NEDQ) [38], or conducting screening interviews. Additionally, future research on NES typology may benefit from testing the validity of NES group categorization, for example, by applying a hierarchical clustering analysis or a latent profile analysis. Moreover, a multinomial logistic regression model may be considered in future research concerning NES subtype grouping and additional variable associations. The current study, however, provides preliminary guidance for using specific items related to evening hyperphagia and nocturnal ingestions to assess NES subtypes. Lastly, another strength is that the hypothesis formulation was based on a comprehensive literature review of NES typology and how binge eating and food addiction symptoms relate to NES symptomology. The results provide novel insights regarding NES latent subtypes and implications on weight-related complications.

## What is already known on this subject?

In comparison to other abnormal eating behaviors, evening hyperphagia and nocturnal eating have received diminutive attention in eating pathology research. Given the distinctive characteristics of evening hyperphagia (i.e., consuming more than 25% of total daily caloric intake after the evening meal) and nocturnal eating (i.e., consuming food upon nocturnal awakenings), studying potential differences associated with three NES subtypes on the basis of evening hyperphagia and/or nocturnal ingestions has been advised [3]. Since, only one study has examined these NES subtypes and the results revealed some differences in sleep and eating patterns between an evening hyperphagia with nocturnal ingestions group and a nocturnal ingestions-only group [22], thus additional research on NES subtypes is warranted. Furthermore, additional research is needed to further predict NES subtype group membership. This will also shed light onto the pathology of NES subtypes and may inform treatment interventions by properly identifying individuals who may meet NES criteria.

## What this study adds?

To the authors’ knowledge, this is the first study to examine differences in binge eating and frequency of food addiction symptoms among latent NES subtypes using self-reported data. Binge eating and food addiction symptoms were significantly more severe and common among participants who fell within an NES subtype encompassing both evening hyperphagia and nocturnal ingestions than participants who only reported one of these criteria. These results highlight the complexity of disordered eating and the importance to conduct well-rounded assessments when targeting behavioral and psychological characteristics of disordered eating. The current study informs future research concerning NES typology and provides direction for future research on the subject.

## Conclusion

NES typology is a relatively novel topic and one that requires further investigation. In the current study, three possible NES subtypes—a combined evening hyperphagia and nocturnal ingestions (EHNI) subtype, an evening hyperphagia-only (EHO) subtype, and a nocturnal ingestions-only (NIO) subtype—were analyzed for eating pathology differences. The results suggest that individuals who fall within an EHNI subtype are at a higher risk of experiencing other, more severe eating pathology, thus potentially being at higher risk for developing obesity. However, contrary to expectations, there were no significant differences in terms of binge eating symptoms between the EHO and NIO groups. Similarly, even though the NIO group’s average for food addiction symptoms was slightly higher than the average of the EHO group (in the hypothesized direction), the mean difference did not reach statistical significance. In addition, the current study aimed to inform current treatment for obesogenic behavior and provide some guidance in clinical assessment and treatment as well as directions for future research of NES typology.

## Data Availability

The datasets generated during and/or analyzed during the current study are available from the corresponding author on reasonable request.

## Footnotes

1 Given the bivariate relationship with study variables, participant age, BMI, and sex were entered in as covariates. Two analyses of covariance (ANCOVAs) were conducted between the three groups on binge eating and food addiction. While both models were significant, *F*(2,160) = 11.59, *p* < .001 and *F*(2,159) = 14.64, *p* < .001, BMI was the only significant covariate (in the food addiction analysis only); *F*(1,159) = 4.64, *p* = .03. No other covariates were significant predictors in either model and given that the results remained unchanged when BMI is included in the food addiction analysis, the original ANOVAs were presented to maintain parsimony and to reflect the original, a priori hypotheses.
